# Effect and Mechanism of Polyethylene Glycol (PEG) Used as a Phase Change Composite on Cement Paste

**DOI:** 10.3390/ma15082749

**Published:** 2022-04-08

**Authors:** Chunpeng Zhang, Chaoming Pang, Yunrui Mao, Zhiyuan Tang

**Affiliations:** Jiangsu Key Laboratory of Civil Engineering Material, School of Materials Science and Engineering, Southeast University, Nanjing 211189, China; 220192007@seu.edu.cn (C.Z.); 15951655570@163.com (Y.M.); 220192055@seu.edu.cn (Z.T.)

**Keywords:** phase change materials (PCM), cement hydration, polyethylene glycol (PEG), mechanism, mechanical properties

## Abstract

The use of phase change materials (PCMs) in the construction industry is one of the primary strategies for addressing the building industry’s present excessive energy usage. However, since PCMs must be enclosed before being used in construction, their efficiency is limited and their compatibility with concrete is poor. Thus, polyethylene glycol (PEG), a sequence of PCMs that may be put directly into concrete, is the target of this research. The fluidity, mechanical properties, thermal properties, hydration process, and hydration products of PEG-600 cement slurry were examined by TAM, XRD, FTIR, DSC, MALDI, etc., methods in this study. Furthermore, we tested the thermal properties of PEG-800 to confirm that the same depolymerization of PEG occurred in an alkaline environment. When PEG, with a molecular weight of 600 (PEG-600), dose was increased to 10%, both compressive and flexural strength fell by 19% and 18%, respectively. The phase change points of both PEG-600 cement paste and PEG-800 cement paste decreased to 10~15 °C, and the enthalpy of the phase change was about 6 J/g. Additionally, it was discovered that PEG entered the reaction during the hydration step. PEG underwent depolymerization and subsequently formed a complex with Ca^2+^. However, due to the large dose of PEG used in this investigation, a self-curing effect of PEG in concrete was not seen. The findings of this research suggest a novel use for PCMs: PEG may be directly applied to concrete to fulfill both mechanical and thermal requirements. Additionally, the number of hydration products and phase compositions remained almost constant.

## 1. Introduction

According to CABEE (Professional Committee on Building Energy and Emissions) research, building energy consumption accounts for 51.3% of China’s total carbon emissions, making it a major priority to reduce building energy [1]. Zero-energy building design is one of the most successful strategies for the sustainable growth of the construction industry, and the use of phase change materials in buildings is a critical component of this.

Phase change materials (PCMs) are a novel kind of green energy material that leverage the endothermic and exothermic effects of phase change to accomplish energy storage and autonomous release, as well as energy utilization [2]. When applied to a building’s external wall, the heat absorbed at a high temperature limits the amount of heat transported to the outer wall through latent heat in the wall. When the temperature is dropped, the heat exchanger returns the stored heat to the environment, providing thermal insulation and minimizing interior temperature swings. Thus, human comfort will be greatly enhanced, and the rate of heating will be lowered if a fire arises, thus slowing the spread of the fire [3,4,5]. PCMs are typically packed first to minimize the potential of leaking. Although these products have a high degree of cyclic stability, their thermal characteristics sometimes diverge from those of PCMs [6,7,8]. However, the package has the potential to mitigate the effect of PCMs on the mechanical characteristics of the concrete. The strength of phase change concrete is dramatically reduced during the mixing of the PCMs, particularly when the PCM dose is increased. Additionally, high-density polyethylene (HDPE) is sometimes used to form paraffin. This approach does not alter the thermal characteristics, although its reliability is questionable [9,10]. The poor thermal conductivity of HDPE/PCM results in lower overall energy storage efficiency, so it is not now widely used.

**Figure 1:**
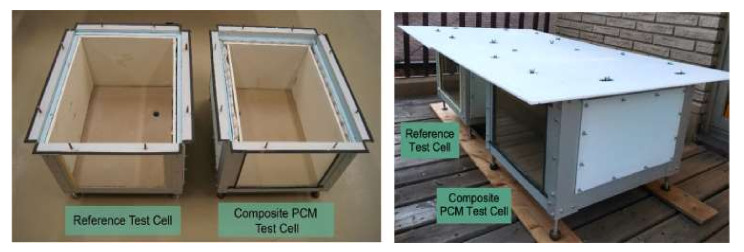
Experimental unit in the study of Ahmad.

Due to its more suitable phase transition point, at about 20~30 °C, and inexpensive cost, polyethylene glycol (PEG) is one of the most often used PCMs in the construction sector. PEG is often coupled with other porous materials to form stable phase change energy storage technologies. The polyethylene glycol/diatomite system has a maximum mass concentration of 58%, a phase transition temperature of 45~57 °C, and a phase transition enthalpy of 91.72~105.70 J/g [11]. Similarly, polyethylene glycol/silica is a popular phase change energy storage material with a maximum mass concentration of 98% and a phase change enthalpy of up to 164.9 J/g [12,13]. However, these fixed PCMs have a low thermal conductivity, which is incompatible with the energy storage function. Currently, the most popular solution to the conductivity issue is to employ in situ copper to replace the shell materials or to augment the thermal conductivity of the device using graphite, graphene, or another high-thermal-conductivity material [14,15,16]. However, the strength of concrete encapsulated with PEG has been found to be dramatically reduced [17]. To maximize energy storage, researchers are also experimenting with directly incorporating phase change energy storage material into the wall interlayer or wall. For instance, PEG-600 was applied directly to a hollow aluminum tube as PCMs to create a phase change energy storage package. Then, it was reinforced with fiber and plywood to create a wallboard. As seen in Figure 1, the wall panels were directly connected to form a basic cube structure. It was discovered that, by using phase change energy storage materials, the temperature fluctuation range in summer could be reduced by more than 20 °C, but the inside temperature may also be enhanced by around 6 °C [18]. Similarly, a pipe filled with PCMs was utilized in the building of mass concrete to lower the temperature increase during the hydration phase of the concrete [19,20,21]. As a result, several researchers have concluded that the direct application of PCMs to building walls or the construction sector is a worthwhile endeavor. Direct application of paraffin to concrete may increase its impermeability and several other properties of durability [22].

PEG with a molecular weight of 400 (PEG-400) and PEG with a molecular weight of 600 (PEG-600) are significant self-curing agents in concrete. When the PEG-400 content is kept within 2.4%, it was discovered that the strength of the concrete may be raised by more than 40%, and the mass-loss rate may be lowered from 4.2% to 2.06% [23]. Another study suggested that PEG-400 may boost strength and fluidity at concentrations ranging from 0% to 6% [24]. PEG-600 can also be used as a self-curing agent in concrete. The compressive strength of concrete with PEG-600 at 28 days is nearly the same as concrete with PEG-400. PEG with a higher molecular weight can also play the role of a self-curing agent in concrete. However, it was found that, as the molecular weight increased, the optimum dosage decreased [25]. In another study, it was found that the optimum dosage of PEG-600, as an internal curing agent, was about 1% with an increase of 23.94% in compressive strength, 20.28% in splitting tensile strength, and 60.98% in the modulus of elasticity over comparative specimens [26]. The mechanism of concrete formation is described as follows: “from the concrete surface, the evaporation of moisture takes place continuously due to the variation in the chemical potential and the free energy space between the liquid and the vapor phase. At the time of mixing the polymers are added to the concrete mainly forming hydrogen bonds between the water molecules; this helps to reduce the chemical potential of the molecules due to reduction in the vapor pressure and in the rate of evaporation from the exterior surface” [27]. Additionally, the nanostructure and the physical and chemical characteristics of C-S-H-PEG are discussed. It has also been found that PEG may alter the structure of C-S-H under specific circumstances, compromising the concrete’s durability [28]. It is important to note that alcoholic hydroxyl groups have a stronger effect on the rate of cement hydration. For example, when the concentration of ethylene glycol is less than 0.3%, it may act as an accelerator of coagulation; however, when the concentration surpasses this threshold, ethylene glycol acts as a retarder. Glycerol and other compounds create comparable difficulties.

Most studies on concrete with PEG as a self-curing agent have focused on strength development and durability changes, while ignoring the thermal property changes brought about by PEG itself as a kind of PCM. Moreover, the research on phase change energy storage of PEG has ignored its excellent compatibility with concrete and encapsulation by various methods. As a self-curing agent, the amount of PEG added directly to the concrete is not more than 2%. In contrast to most available research, this study examined the maximum quantity of PEG-600 in the cement paste while also examining the development of the cement paste’s mechanical characteristics and porosity at PEG contents of 0% to 10%. It further examined the microstructure of PEG-600 that exists in cement paste at various doses and water–cement ratios, as well as its effect on energy storage, the cement hydration process, and the hydration products. Following the above investigation, this study concludes that PEG has significant research value as a kind of PCM that may be directly added to concrete and has only minor adverse impacts on the cement matrix.

## 2. Materials and Methods

### 2.1. Materials

Portland cement P·II52.5, which was produced by Jiangnan Xiaoyetian Cement Co., Ltd. (Nanjing, China), was used in this study. The chemical and physical characteristics of the cement are shown in Table 1 and Table 2.

Generally, PEG-600 and PEG-800 were used in this study. The vendors and types of PCM are shown in Table 3. Tap water was used as the mixing water throughout the study.

### 2.2. Sample Preparation

Sand and stones affect many microscopic tests, such as XRD. Therefore, cement paste was used for all experiments in this study.

All PCMs were kept warm at 30 °C for about 8 h so that the PCMs were all stable during the process of forming. The liquid PCM was first mixed with water and fully stirred for 1 min and was then subject to further mixing. The solid was first mixed with powder and fully stirred for 1 min and then water was added to the mix. After that, the specimens were molded and then routinely cured at 20 °C and 95% R.H.

For the cement paste specimens, after testing of 28 d compressive strength, the hydration of paste samples was terminated with isopropyl alcohol. After drying, the samples were crushed and ground into powder immediately before the relevant test.

### 2.3. Methods

#### 2.3.1. Strength and Fluidity Test

The compressive strength and flexible strength of the cement paste were tested according to GB/T 17671-1999. Three prismatic specimens, with dimensions of 40 mm × 40 mm × 160 mm, were prepared at each specific experimental age, and, to begin with, the flexural strength was tested at a speed of 50 ± 10 N/s. Then each specimen was broken into two specimens, for a total of 6 specimens, for compressive strength testing at a speed of 2400 ± 200 N/s. The fluidity of the cement paste was tested using a truncated cone with a size of 36 mm at the upper bottom, 60 mm at the lower bottom, and of 60 mm height, according to GB/T 8077-2005,

#### 2.3.2. Analysis of Cement Hydration Heat (Isothermal Calorimetry)

The hydration heat of the cement paste was measured using a TAM air thermostat calorimeter made by the American TA Company. After mixing the cement-based material mixture to be tested, it was added into the sample ampoule and immediately put into the constant temperature calorimeter to start the measurement. During the test, the temperature of the sample was strictly controlled to be the constant temperature required for the test. The cumulative heat release of cement hydration for 72 h was continuously measured, and the heat release rate curve was further analyzed.

#### 2.3.3. X-ray Diffraction Analysis

The crystalline phases of the PCM–cement paste were characterized by X-ray diffraction (XRD). An X-ray diffractometer (X’Pert3 Powder, Malvern Panalytical, Malvern, UK) was used with measurement conditions of CuKα radiation and 2-theta scanning from 5° to 60°, with a scan speed of 0.15° per second. About 3 g of powder was used in the XRD test.

#### 2.3.4. Differential Scanning Calorimetry Analysis

This experiment used a DSC8000 differential scanning calorimetry analyzer produced by the PE company (Boston, MA, USA). The temperature range used in the test was −75 °C to 550 °C, with a temperature accuracy of 0.05 °C and a temperature rise rate of 5 °C/min, with a temperature accuracy of 0.008 °C and a sensitivity of 0.18 mW.

#### 2.3.5. Fourier Transform Infrared Spectra Analysis

Fourier transform infrared (FTIR) spectra of the PCM itself and the cement paste containing PCMs were recorded from 4000 to 400 cm^−1^ with a resolution of 4 cm^−1^ using a Thermo Fisher Nicolet IS50 FTIR spectrometer produced by Thermo Fisher Scientific (Waltham, MA, USA). The test samples were prepared as a powder, as shown in Section 2.2.

#### 2.3.6. Matrix-Assisted Laser Desorption/Ionization

Matrix-assisted laser desorption/ionization (MALDI) is a mild ionization technique for mass spectrometry, which can obtain mass spectrometry information for complete macromolecules that are easily dissociated into fragments by conventional ionization methods. MALDI spectra of the PCMs with NaOH or Ca(OH)_2_ were recorded to obtain the relative molecular masses of the PCMs after reaction in the alkali environment. PEG-600 was mixed with CaCl_2_ to keep the concentration of Ca^2+^ the same in the NaOH and Ca(OH)_2_ environments. The pH value of PCM with NaOH was 13 and the pH value of PCM with Ca(OH)_2_ was 11.

## 3. Results

### 3.1. Maximum Amount of PEG-600 in the Cement Matrix

**Figure 2:**
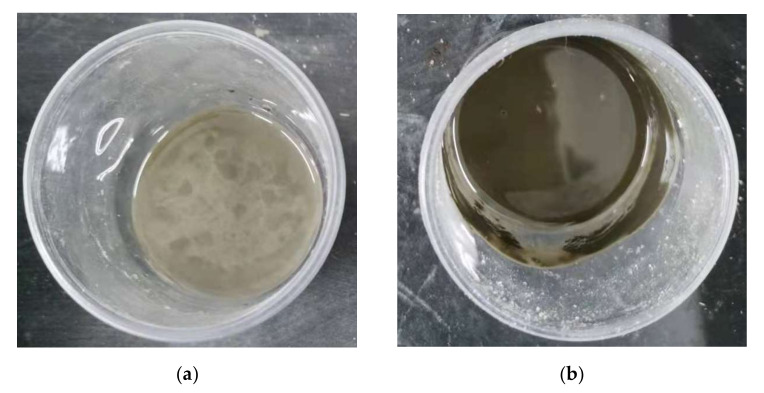
(**a**) Cement paste (water ratio 0.4) with 50% PEG-600; (**b**) cement paste (water ratio 0.3) with 50% PEG-600.

As shown in Table 4, when the dosage of PEG-600 exceeded 10%, the setting time increased significantly. Additionally, it was discovered that increasing the water–cement ratio decreased the setting time when the same amount of PEG-600 was used. When the dosage of PEG-600 was greater than 40%, the cement paste did not hydrate at all, even after more than half a year. This phenomenon is similar to that for other organic substances containing alcoholic hydroxyl groups, such as ethylene glycol or glycerol. As shown in Figure 2, PEG-600 has a high water absorption capacity and is completely miscible with water. Thus, PEG-600 can first combine with water during the hydration process and then form a water film, preventing sufficient water from participating in the cement hydration reaction. Calcium can also react with alcoholic hydroxyl groups to form calcium alkoxide, which is toxic to calcium and impairs progression of the hydration reaction. As a result, the average water–cement ratio used in the subsequent research design of this study was 0.4–0.5, and the amount of PEG-600 was 0–10%.

PEG-600 significantly improved the fluidity of the cement. When the cement paste’s fluidity was excessive, it was difficult to install molds and easy to segregate bleeding. As shown in Table 5, the fluidity of the net pulp exceeded the testable range at a water—cement ratio of 0.5 and a PEG-600 dosage of 0.10, indicating that this mix ratio was inapplicable.

Ultimately, as shown in Table 6 below, the following seven mix ratios were selected for follow-up research.

### 3.2. Effect of PEG-600 on the Strength of Cement Paste

**Figure 3:**
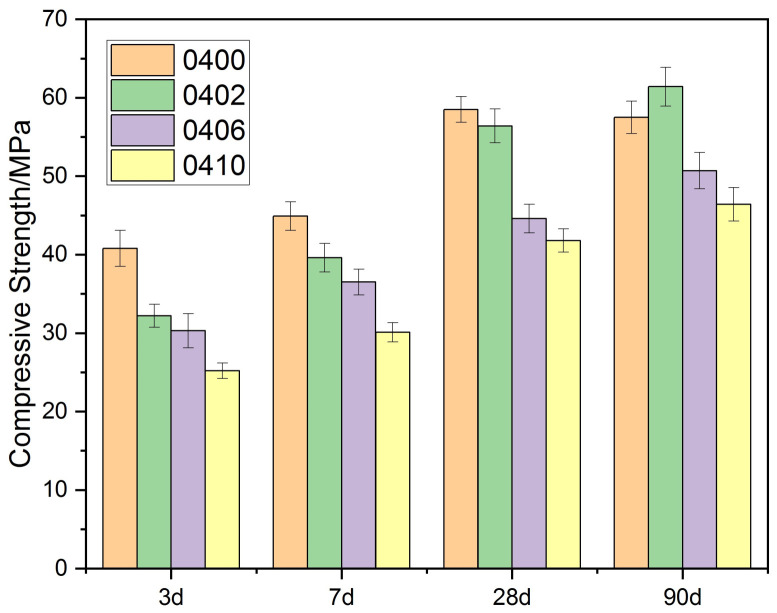
Compressive strength of cement paste (W/C = 0.4) with PEG-600.

**Figure 4:**
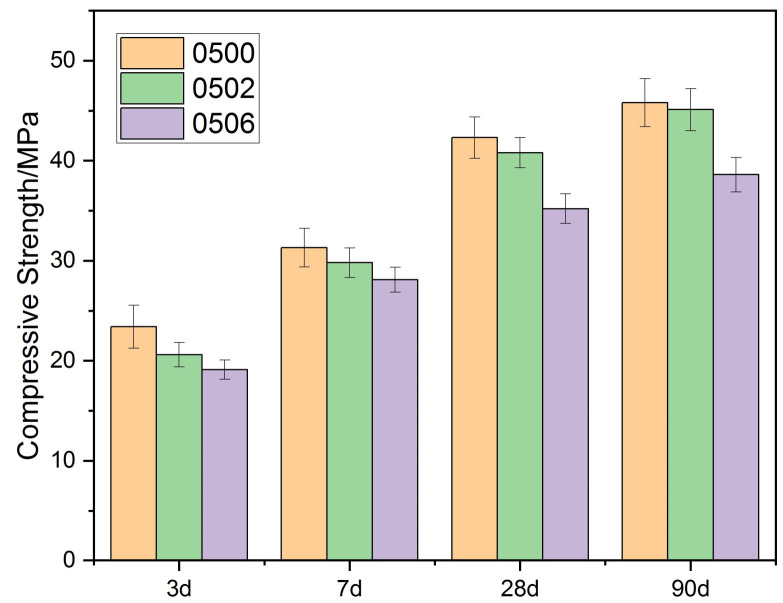
Compressive strength of cement paste (W/C = 0.5) with PEG-600.

As shown in Figure 3 and Figure 4, the compressive strength of each group’s cement paste gradually decreased as the PEG-600 dosage was increased. When the PEG-600 dosage was increased to 10%, either in a 0.4 or a 0.5 water–cement ratio, the compressive strength of the two groups decreased by 19% and 16%, respectively. When PEG-600 was used at a 2% concentration, it was discovered that the compressive strength of the specimens decreased slightly when compared to the control group. When the water/cement ratio was 0.4, the specimens’ strength decreased by 3.59%; when the water–cement ratio was 0.5, the specimens’ strength decreased by 3.55%. According to the IJREA series of studies, PEG-600 can also play a role in promoting the hydration process and was shown to increase the strength of concrete by 5.7% in internal curing at 1.0% [26]. In another study, the compressive strength with PEG of 1.5% increased by 23.9% [25]. Combining this with the internal curing mechanism of materials that absorb water in the pores represented by ceramsite, it was discovered that the pore size distribution of ceramsite had a significant effect on the material’s water absorption performance. Since ceramsite has a high capacity for water absorption, it will not release water during the hydration process. The situation was similar for the PEG series with varying degrees of polymerization and varying dosages. The amount of absorbable water increases with increase in PEG doping. As a result, it becomes nearly impossible to release the absorbed water, and the internal curing effect diminishes and is eventually supplanted by negative effects.

**Figure 5:**
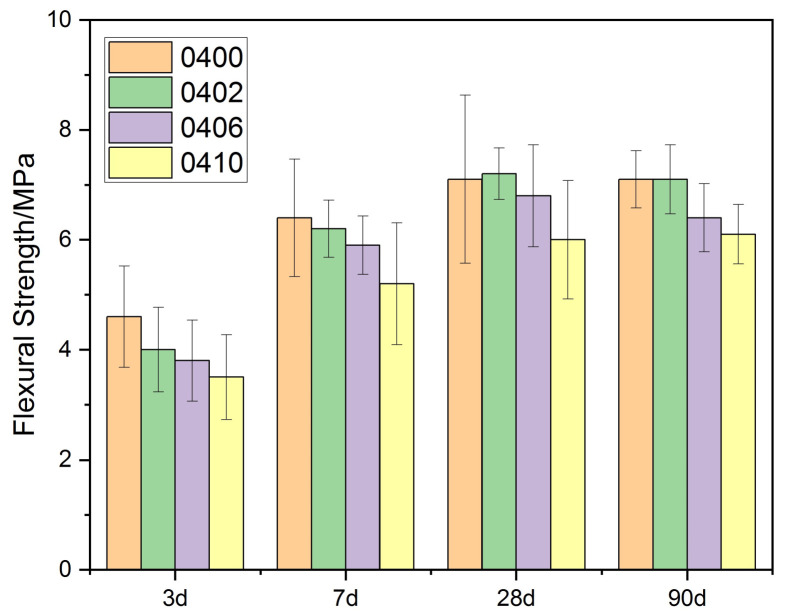
Flexural strength of cement paste (W/C = 0.4) with PEG-600.

**Figure 6:**
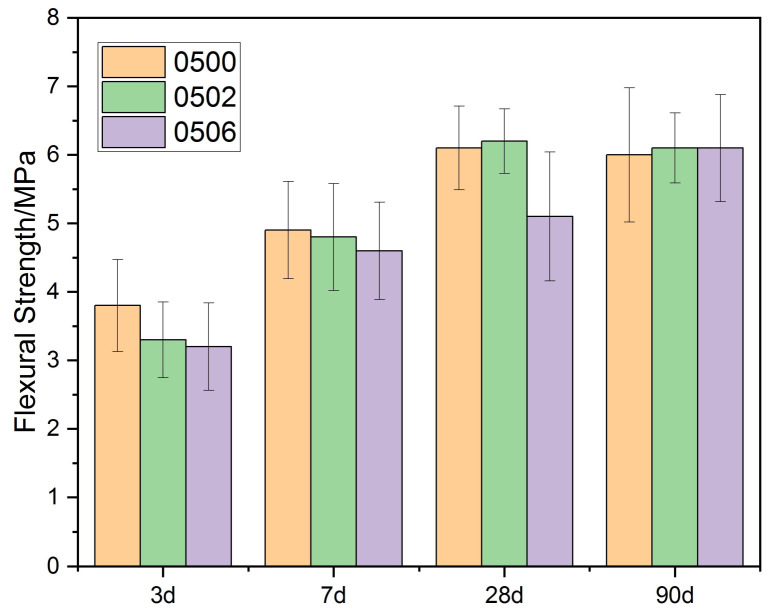
Flexural strength of cement paste (W/C = 0.5) with PEG-600.

As shown in Figure 5 and Figure 6, the flexural strength of each cement paste increased initially and then decreased as the PEG-600 dosage was increased. When PEG-600 was used in small amounts, it increased the flexural strength of the cement matrix by approximately 2%. However, increasing the PEG-600 dosage impaired the cement hydration process in the low water–cement ratio group, resulting in a decrease in overall flexural strength. When the PEG-600 dosage was increased to 10%, the specimens’ flexural strength decreased by 18%. However, the flexural strength of the group with a high water/cement ratio did not significantly decrease. When the PEG-600 dosage was increased to 10%, the 90 d flexural strength did not decrease. As with compressive strength, it was found that water absorption did not have a significant effect on flexural strength. Additionally, a higher water/cement ratio was found to mitigate the impact.

It should be emphasized that the decrease in 28 d strength, as well as in 56 d strength, was around 50% when PCM with microencapsulation or PCM with lightweight aggregate penetration was added to the concrete with a strength of about 50 MPa [29]. When the PEG/SiO_2_ system was added to the concrete, the 28 d strength of the concrete decreased from 54.4 MPa to 35.3 MPa at a 10% volume admixture, which was about 35% [30]. In summary, the direct addition of polyethylene glycol to cementitious materials had a much smaller effect on strength.

### 3.3. Effect of PEG on the Thermal Properties of Cement Paste

As shown in Table 7, the endothermic and exothermic peaks had peak temperatures of 13 °C and 26 °C, respectively, indicating that the PEG itself had a high degree of supercooling, which is unfavorable for its use in building materials. However, when compared to PEG before the addition of the cement paste, the supercooling degree of PEG in the cement matrix was significantly reduced, even to 1 °C, significantly increasing its application efficiency. It is worth noting, however, that even with varying degrees of PEG polymerization, the phase transition point in the cement matrix was nearly identical, and that the phase transition points were significantly reduced when compared to the original PEG. In an alkaline environment, PEG underwent depolymerization, and as the degree of polymerization of PEG decreased, the phase transition point of PEG decreased as well.

The phase change enthalpy of the PEG cement paste was about 6 J/g. This was because the calculation procedure for the phase change enthalpy of the PEG cement paste took into account the sum of the polyethylene glycol and concrete masses. Similarly, the phase change enthalpy of the PEG/SiO_2_ concrete was only 4 J/g, if the concrete was calculated together with the encapsulated PEG mass, while the theoretical phase change enthalpy of concrete is only about 6.5 J/g for the same mass fraction as in this study for the case of the highest encapsulation efficiency previously mentioned [12,13,30]. Therefore, the phase change enthalpy loss of polyethylene glycol added directly to concrete was close to the effect of first encapsulation and then addition.

The decomposition products of PEG in cement were analyzed. As shown in Table 6, the phase transition temperatures of PEG with varying degrees of polymerization in the cement matrix were exactly equivalent. It can be concluded that PEG with varying degrees of polymerization will depolymerize to a similar degree of polymerization following hydration. However, PEG with low polymerization degrees (PEG-L) exhibited a low phase transition temperature. Thus, during the hydration process, PEG-L may react with calcium to form a calcium complex between calcium and PEG [31].

As shown in Table 8, PEG depolymerizes in a strongly alkaline environment, and the product has a similar molecular weight. PEG undergoes irregular chain breaking in the presence of strong oxidizing agents, hydroxyl groups and other free radicals to produce PEG with low polymerization [32]. The degree of polymerization of the PEG decomposition products was comparable to that in the sodium hydroxide environment, indicating that the alkalinity of the saturated calcium hydroxide was sufficient to decompose PEG completely. It can be deduced that PEG can be depolymerized in the cement matrix to form a shorter-chain PEG. However, PEG-n has a molecular weight of 44.05n + 18.02 g/mol, which is greater than the molecular weight we detected. PEG can form a molecular chain between calcium ions and its ionic ratio is approximately six PEG structures per calcium ion. Its structure resembles that of octadecone [31]. However, another study reported that PEG can react with CaCl_2_ and form a complex with four PEG structures [33]. PEG complexed with calcium alters the phase transition point in conjunction with the depolymerization process.

### 3.4. Effect of PEG-600 on Cement Hydration

#### 3.4.1. Effect of PEG-600 on the Early Process of Cement Hydration

**Figure 7:**
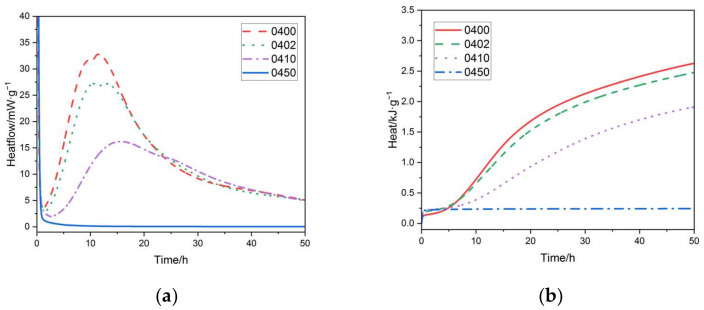
(**a**) Rate of hydration heat release of cement paste (W/C = 0.4) with different content of PEG-600. (**b**) Hydration heat curves of cement paste (W/C = 0.4) with different content of PEG-600.

**Figure 8:**
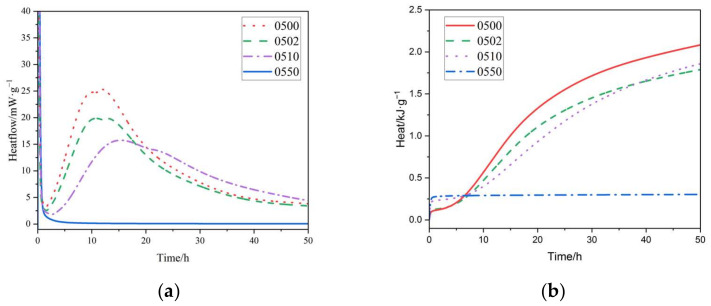
(**a**) Rate of hydration heat release of cement paste (W/C = 0.5) with different content of PEG-600. (**b**) Hydration heat curves of cement paste (W/C = 0.5) with different content of PEG-600.

As shown in Figure 7 and Figure 8, increasing the PEG-600 dosage resulted in a decrease in the hydration heat of cement at both water–cement ratios. The first peak of the heat of hydration curve, the heat of dissolution exothermic peak, occurred significantly higher. In our study, polyethylene glycol was added to the cementitious material after it was fully mixed with water, so the exothermic heat of the intercalation process between PEG and water could be excluded. This process occurs due to the reaction between polyethylene glycol and calcium in the cementitious base in an alkaline environment. When the PEG-600 dosage of the cement paste approached 50%, hydration did not occur at both 0.4 and 0.5 water–cement ratios. This was consistent with the results of the previously set up time tests. When a sufficiently high dose of PEG-600 was used, the cement hydration process completely halted. Increasing the PEG-600 concentration had a similar effect on the peak shaving and delay effects of the heat of cement hydration at various water–cement ratios. Both water/cement ratio specimens, however, exhibited a significant decrease in this section. This was in contrast to the results of the set-up time test. Due to the low PEG-600 concentration, the total dosage was low, and there was little competition for water for cement hydration. All groups that included PEG-600 generated significantly more heat than other groups during the hydrolysis stage, owing to the intense heat generated by PEG-600′s depolymerization process in an alkaline environment. Based on long-term strength data, it was determined that the peak shaving and delay effects of an acceptable amount of PEG-600 on cement hydration do not impair long-term performance significantly.

#### 3.4.2. Effect of PEG-600 on Cement Hydration Products

**Figure 9:**
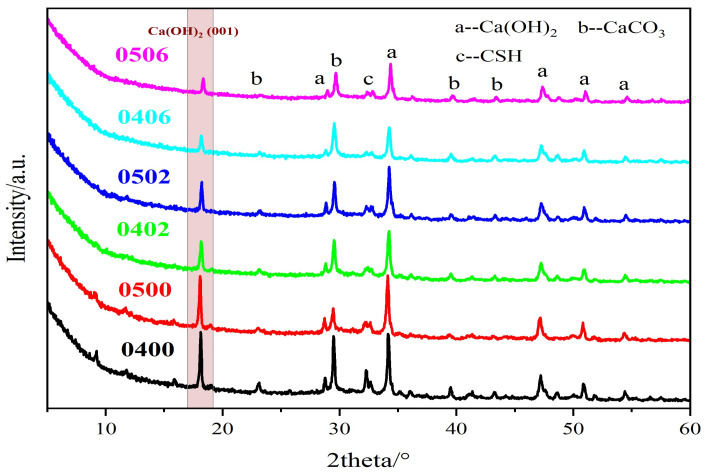
XRD of cement paste with PEG-600 from 0% to 6%.

As shown in Figure 9, there were no significant changes in the XRD peaks of cement paste with PEG-600 and without PEG-600, except for the 17~19 nm region, where the crystal interplanar spacing was about 4.90 nm. This part is usually regarded as the characteristic peak of CH, exactly at the (001) planes of CH. With increasing dosage of PEG-600, the CH peak decreased dramatically.

According to the nucleation and growth theory, the free energy of two-dimensional nucleation is much smaller than the three-dimensional free energy [34], so that, when the degree of supersaturation is not enough to form a three-dimensional nucleus, a two-dimensional nucleus will be formed. According to the results of theoretical calculations, the maximum degree of calcium hydroxide saturation in Portland cement is only 0.36, which is maintained at a low level throughout the life cycle [35]. It was also found that both impurity ions and the degree of calcium hydroxide supersaturation have a huge effect on the {001} family of crystal planes [36]. PEG-600 did not contain any impurity ions, such as Na^+^, Cl^−^ or SO_4_^2−^, so it can be confirmed that PEG-600 reacted with calcium. During the process of depolymerization, the phase change point of PEG decreased significantly. However, the phase change point of the complex of Ca and PEG was much larger than for PEG itself. The melting point of PEG, whose molecular number is less than 200, was about −13 °C, but after the complex reaction with Ca^2+^, it increased significantly [31].

To further confirm whether the calcium in the cement matrix is poisoned by alcoholic hydroxyl groups, FTIR tests were carried out. To differentiate the PEG in liquid samples from cement paste and PEG cement paste in solid samples, the absorbance and transmission of the FTIR spectrum were used as the ordinates.

**Figure 10:**
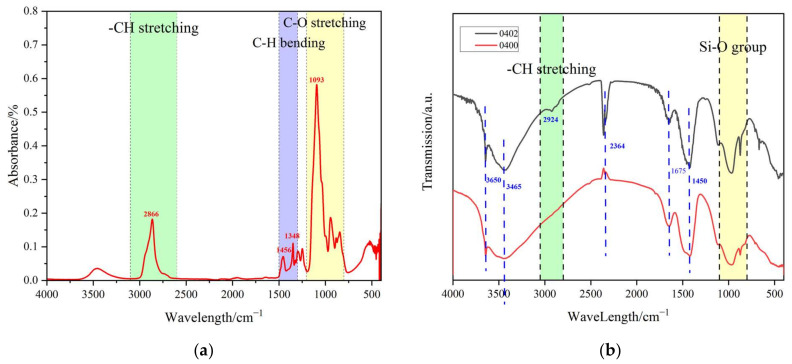
(**a**) FTIR spectra of PEG-600. (**b**) FTIR spectra of cement paste (W/C = 0.4) with PEG-600 0% to 2%.

As shown in Figure 10, there was one special absorption peak compared to the control cement matrix and pure PEG-600. Due to the -CH stretching in PEG-600, when PEG-600 was added to the cement paste, the peak at 2924 cm^−1^ was attributed to -CH stretching in PEG-600. Calcium hydroxide had a characteristic peak at 3650 cm^−1^, which represents the stretching vibration of O-H [37]. Further there was another peak at 3465 cm^−1^, which also represented the stretching vibration of O-H. There was also a peak at 1675 cm^−1^, so it can be inferred that the powder contained water [38]. The absorption peak at 2363 cm^−1^ corresponded to CO_2_ in air [39,40,41]. The characteristic vibrational peaks of carbonate appeared at 1450 cm^−1^ [42]. After the complexation reaction between PEG-600 and Ca, the peak of the C-O bond in PEG-600 shifted in the wavelength range of 1000–1100 cm^−1^ [31]. However, the absorption peak of the Si-O bond was stronger than that of the C-O bond, so this phenomenon could not be observed. Therefore, further experimental exploration is required.

## 4. Conclusions

Polyethylene glycol was directly added to the cement matrix, and it still retained a certain degree of phase changeability, and, at the same time, there was no leakage. The following conclusions were drawn for our study:

(1) PEG significantly improved the cement matrix’s fluidity. When the content reached 10%, the fluidity increased by 35% at a water–cement ratio of 0.4 and exceeded the measurement limit at a water–cement ratio of 0.5. The strength of cement paste decreased as the PEG dosage increased. When the PEG content increased to 10% under w/c ratios from 0.4 to 0.5, the compressive strength decreased by about 20%, while the flexural strength decreased by 18% and 0%, respectively. It can be inferred that the addition of PEG had a limited effect on the cement matrix’s strength.
(2) PEG can significantly slow down the process of cement hydration, according to research on the heat of hydration. When the blending percentage of PEG reached 50%, the hydration reaction completely stopped. Additionally, it was discovered during the setting time test that the PEG could significantly delay the hydration process. The XRD analysis revealed that the (001) face of Ca(OH)_2_ in the cement paste was quite small. Thus, the primary reason for these phenomena was PEG’s toxicity to Ca^2+^ and the absorption of water.
(3) The PEG phase transition point decreased to 10~15 °C in the cement matrix, and the enthalpy of the phase change decreased slightly. The FTIR spectra indicated that PEG reacted with the cement during the hydration process. In comparison to the control group, a distinct peak corresponding to Ca(OH)_2_ could be identified. Additionally, the molecular weight of the PEG in a strongly alkaline environment containing Ca^2+^ indicated that the PEG had depolymerized and formed a complex with Ca^2+^.
(4) While retaining a certain amount of phase change energy storage, the higher doping of PEG had no serious negative effects. Therefore, other thermal properties of this system should be further explored and studied, such as leakage, thermal performance, cyclic stability, and the durability of PEG-cement composites.

## Figures and Tables

**Table 1:** Physical properties of cement.

Items	Setting Time/min		Flexural Strength/MPa		Compressive Strength/MPa	
	Initial	Final	3 d	28 d	3 d	28 d
Results	199	285	6.6	7.2	34.1	53.2

**Table 2:** Chemical properties of cement.

Oxide	SiO_2_	Al_2_O_3_	Fe_2_O_3_	CaO	Na_2_O	K_2_O	SO_3_	MgO	Cl (ppm)
Wt%	22.41	3.26	0.12	68.81	0.27	0.16	3.27	1.70	290

**Table 3:** Properties of phase change materials provided by the vendor.

Name	Vendor	Type	Freezing Point/°C	Molecular Weight	pH Value
PEG-600	SINOPHARM	Liquid	18~22	570~630	5.0~7.0
PEG-800	SINOPHARM	Solid	26~30	720~880	5.0~7.0

**Table 4:** Effect of PEG-600 dosage and water–cement ratio on setting time of cement matrix.

	PEG Dosage	0%/h	10%/h	20%/h	30%/h	40%	50%
W/C							
0.3		<12	<24	<48	<108	Without setting	Without setting
0.4		<12	<24	<36	<84	Without setting	Without setting
0.5		<12	<24	<24	<60	Without setting	Without setting

**Table 5:** Effect of PEG-600 dosage and water–cement ratio on the fluidity of cement paste.

	PEG Dosage	0%/mm	2%/m	6%/mm	10%/mm
W/C					
0.4		180	185	200	235
0.5		220	225	245	>300

**Table 6:** Mix ratio of the cement paste used in the test.

Number	W/C	Water/g	Cement/g	PEG-600/g	PEG-600/%
0400	0.4	40	100	0	0
0402	0.4	40	100	2	2
0406	0.4	40	100	6	6
0410	0.4	40	100	10	10
0500	0.5	50	100	0	0
0502	0.5	50	100	2	2
0506	0.5	50	100	6	6

**Table 7:** Phase transition point of PEG cement paste with different degrees of polymerization.

Degree of Polymerization of PEG	Original Phase Change Point by Vender/°C	Original Phase Change Point by Test/°C	Phase Change Point of Cement-Based PEG/°C	Original Phase Change Enthalpy /J·g^−1^	Phase Change Enthalpy with Concrete/J·g^−1^
600	20 ± 2	13~26	13.5 ± 1	117	5.9601
800	28 ± 2	22~36	10.5 ± 1	110	6.0047

**Table 8:** Molecular weight of PEG depolymerized in a strong alkaline environment.

Conditions	Tolerance	Minimum	Maximum
PEG-600	~44	25,126.52	27,769.52
Sodium hydroxide	~44	525.400	1318.076
Calcium hydroxide	~44	525.454	1318.071
